# Therapeutic Potential of Exosomes Derived from Adipose Tissue-Sourced Mesenchymal Stem Cells in the Treatment of Neural and Retinal Diseases

**DOI:** 10.3390/ijms23094487

**Published:** 2022-04-19

**Authors:** Carl Randall Harrell, Vladislav Volarevic, Valentin Djonov, Ana Volarevic

**Affiliations:** 1 Regenerative Processing Plant, LLC, 34176 US Highway 19 N, Palm Harbor, FL 34684, USA; dr.harrell@regenerativeplant.org; 2 Department of Genetics, Faculty of Medical Sciences, University of Kragujevac, 69 Svetozar Markovic Street, 34000 Kragujevac, Serbia; 3 Department of Microbiology and Immunology, Faculty of Medical Sciences, University of Kragujevac, 69 Svetozar Markovic Street, 34000 Kragujevac, Serbia; 4 Institute of Anatomy, University of Bern, Baltzerstrasse 2, 3012 Bern, Switzerland; valentin.djonov@ana.unibe.ch; 5 Department of Cognitive Psychology, Faculty of Medical Sciences, University of Kragujevac, 69 Svetozar Markovic Street, 34000 Kragujevac, Serbia

**Keywords:** adipose tissue-derived mesenchymal stem cells, exosomes, therapy, neural diseases, retinal diseases

## Abstract

Therapeutic agents that are able to prevent or attenuate inflammation and ischemia-induced injury of neural and retinal cells could be used for the treatment of neural and retinal diseases. Exosomes derived from adipose tissue-sourced mesenchymal stem cells (AT-MSC-Exos) are extracellular vesicles that contain neurotrophins, immunoregulatory and angio-modulatory factors secreted by their parental cells. AT-MSC-Exos are enriched with bioactive molecules (microRNAs (miRNAs), enzymes, cytokines, chemokines, immunoregulatory, trophic, and growth factors), that alleviate inflammation and promote the survival of injured cells in neural and retinal tissues. Due to the nano-sized dimension and bilayer lipid envelope, AT-MSC-Exos easily bypass blood–brain and blood–retinal barriers and deliver their cargo directly into the target cells. Accordingly, a large number of experimental studies demonstrated the beneficial effects of AT-MSC-Exos in the treatment of neural and retinal diseases. By delivering neurotrophins, AT-MSC-Exos prevent apoptosis of injured neurons and retinal cells and promote neuritogenesis. AT-MSC-Exos alleviate inflammation in the injured brain, spinal cord, and retinas by delivering immunoregulatory factors in immune cells, suppressing their inflammatory properties. AT-MSC-Exos may act as biological mediators that deliver pro-angiogenic miRNAs in endothelial cells, enabling re-vascularization of ischemic neural and retinal tissues. Herewith, we summarized current knowledge about molecular mechanisms which were responsible for the beneficial effects of AT-MSC-Exos in the treatment of neural and retinal diseases, emphasizing their therapeutic potential in neurology and ophthalmology.

## 1. Introduction

Detrimental immune response has a prominent role in the development and progression of neuroinflammatory, neurodegenerative, and retinal diseases [1]. Injuries and the invasion of microbial pathogens in the eyes and central nervous system elicit a strong immune response, which induces damage to the blood–brain and blood–retinal barriers and causes extensive loss of neural and retinal cells [1,2]. A massive release of alarmins and self-antigens from damaged cells triggers the transformation of the resident microglia into an activated state [2,3,4]. Activated glial cells produce pro-inflammatory cytokines (tumor necrosis factor-alpha (TNF-α), interleukin (IL)-1β, IL-6, IL-23), and chemokines (monocyte chemoattractant protein 1 (MCP-1), chemokine (C-C/C-X-C motif) ligand (CCL)6, CXCL9, CXCL10), which attract circulating leukocytes in the inflamed regions [3]. Recruited dendritic cells (DCs) capture, phagocyte, and degrade microbial antigens into the small polypeptide fragments which are, within major histocompatibility complex (MHC) molecules, presented and delivered to the naïve CD4+ and CD8+ T cells in the regional lymph nodes [3,4,5]. DC-derived interleukin IL-12 activates Th1 lineage defining transcriptional factors (T-box protein expressed in T cells (T-bet) and signals transducer and activator of transcription (STAT)-4) and induce differentiation of antigen-specific naïve CD4+T cells in effector, interferon-gamma (IFN-γ)-producing Th1 cells [3,4,5]. DC-sourced IL-1β, IL-6, and IL-23 activate Th17 lineage-defining transcriptional factors (RAR-related orphan nuclear receptor gamma T (RORyT) and STAT-3) in naïve CD4+ T cells and induce the generation of IL-17 and IL-22-producing effector Th17 cells. Th1 and Th17 cell-driven immune responses are mainly responsible for the development of inflammation-induced loss of neural and retinal cells [3,4,5]. CD4+Th1 cells-sourced IFN-γ activates inflammatory M1 microglia/macrophages while CD4+Th17 cells produce IL-17 and IL-22 that activate inflammatory N1 neutrophils to produce nitric oxide (NO), reactive oxygen species (ROS), which cause oxidative stress in neural and retinal cells [3,4,5]. Additionally, M1 macrophages/microglia and N1 neutrophils produce a large number of inflammatory cytokines and chemokines, crucially contributing to the aggravation of ongoing inflammation. Therefore, continuous and long-term activation of Th1/Th17 cells, M1 macrophages/microglia, and N1 neutrophils create an “inflammatory loop” that induces permanent and irreversible damage to neural and retinal cells [3,4,5].

In order to prevent excessive tissue injury, immunosuppressive immune cells (alternatively activated M2 macrophages/microglia, tolerogenic DCs, and FoxP3-expressing T regulatory cells (Tregs)) produce anti-inflammatory cytokines (IL-10, IL-35, and transforming growth factor-beta (TGF-β)) and create immunosuppressive microenvironment in inflamed eyes, brain, and spinal cord in order to provide protection to the injured neural and retinal cells [6,7]. Since a weakened and suppressed immune response is incapable of eliminating pathogenic microorganisms, while over-activated immune cells elicit neuroinflammation and cause neurodegeneration, an interplay and balance between pro-inflammatory and anti-inflammatory immune cells is crucially responsible for the restoration of the homeostasis in inflamed eyes and the central nervous system [6,7]. Accordingly, therapeutic agents that are able to regulate immune response and simultaneously provide trophic support to injured neurons and retinal cells could be considered new remedies for the treatment of neurological and retinal diseases.

Adipose tissue (AT) represents a valuable source of mesenchymal stem cells (MSCs) [8]. AT-derived MSCs (AT-MSCs) are adult, self-renewable multipotent cells that possess potent immunoregulatory, angio-modulatory, and neuroprotective properties [8]. Results obtained in a large number of experimental and clinical studies demonstrated that either systemic or local (intracerebral or intraocular) injection of AT-MSCs had beneficial effects in the treatment of neural and retinal diseases [9,10,11,12]. AT-MSCs engraft in injured tissues and produce neurotrophins, angio-modulatory, and immunoregulatory factors that suppress the detrimental immune response and promote regeneration of injured neural and retinal cells [9,10,11,12].

Despite these promising results, there are several safety issues that limit AT-MSC-based therapy [13,14,15]. Firstly, AT-MSCs are not constitutively immunosuppressive cells [13]. AT-MSCs may adopt phenotypes and function under the influence of inflammatory cytokines to which they are exposed. When AT-MSCs engraft in the tissue with low levels of TNF-α and IFN-γ, they obtain pro-inflammatory phenotypes and secrete a large number of bioactive factors, which aggravates ongoing inflammation. On the contrary, when AT-MSCs are exposed to the high levels of TNF-α and IFN-γ, they acquire immunosuppressive phenotypes and produce immunoregulatory factors that suppress the immune response [13]. Since local tissue concentration of TNF-α and IFN-γ differ in different phases of inflammation, there is a concern that AT-MSCs that will be engrafted in the retinal and neural tissues with a low level of TNF-α and IFN-γ may obtain pro-inflammatory phenotypes and may aggravate ongoing inflammation. Additionally, AT-MSCs are not “immune privileged” cells since they express MHC class II molecules [14]. Accordingly, transplantation of allogeneic AT-MSCs may provoke a strong immune response in MHC-miss-matched recipients [14]. Finally, TGF-β and bone morphogenetic proteins, released by macrophages and parenchymal cells in inflamed brain and eyes, may induce spontaneous and unwanted chondrogenic and osteogenic differentiation of transplanted AT-MSCs [15].

AT-MSC-derived exosomes (AT-MSC-Exos) are nano-sized extracellular vesicles that contain all neurotrophins, immunoregulatory, and angio-modulatory factors secreted by their parental AT-MSCs [16,17,18]. As cell-free products, AT-MSC-Exos address all safety concerns related to the transplantation of AT-MSCs [19]. Accordingly, injection of AT-MSC-Exos has been considered an alternative therapeutic approach to AT-MSC-based therapy in the treatment of neural and retinal diseases [16,17,18,19].

## 2. Molecular Mechanisms Responsible for the Beneficial Effects of AT-MSC-Exos

AT-MSC-Exos are cup-shaped extracellular vesicles that express CD105, CD29, CD73, CD44, CD9, CD81, TSG101, Calnexin, and lack expression of CD34, IgG1, IgG2b, CD11b, CD45, molecules that are present on the membranes of endothelial cells (ECs) and leucocytes [20]. Due to their nano-sized dimension and bilayer lipid envelope, AT-MSC-Exos easily bypass blood–brain and blood–retinal barriers and deliver their cargo directly into the ECs, immune cells, and injured neurons and retinal cells [19].

As determined by transcriptome and multi-omics sequencing technologies, AT-MSC-Exos are enriched with bioactive molecules (microRNAs (miRNAs), enzymes, cytokines, chemokines, immunoregulatory, trophic, and growth factors), that are responsible for AT-MSC-Exo-dependent beneficial effects in inflamed and injured neural and retinal tissues (Table 1) [20,21,22,23,24,25,26,27].

AT-MSC-Exos contain a large number of miRNAs that incorporate into the RNA-induced silencing complex and effectively inhibit gene expression [21]. Among more than 170 different miRNAs, which are found in exosomes from different stem cell sources [21], miR-486-5p, miR-10a-5p, miR-10b-5p, miR-191-5p, miR-222-3p, and miR146a were the most frequent miRNAs in AT-MSC-Exos. AT-MSC-Exo-sourced miR-486-5p regulate neurogenesis and brain development [22], miR-10a-5p and miR-10b-5p prevent apoptosis of injured neurons [23], miR-191-5p facilitates cell viability and inhibited apoptosis of microglial cells [24], miR222-3p regulates angiogenesis [25] and miR146a down-regulates the expression of IFN-γ in Th1 lymphocytes [26].

More than 370 proteins were found in AT-MSC-Exos. AT-MSC-Exos contain proteins that modulate neurodevelopment and lymphocyte activation (ADAM9, ADAM10, CACNA2D1, NOTCH2), repair and regeneration of injured tissues (WNT4, PAI-1, matrix metalloproteinase (MMP)-2 and 9) and are enriched in proteins that regulate oxidative stress (ATP2B1, ATP1A1, peroxiredoxin (PRDX)-1,-2,-4,-6) [27]. AT-MSC-Exos also contain neurotrophins (glial cell-derived neurotrophic factor (GDNF)), fibroblast growth factor-1 (FGF-1), brain-derived neurotrophic factor (BDNF), insulin-like growth factor-1 (IGF-1) and neural growth factor (NGF)), which provide trophic support to injured neurons and promote axonal regeneration [27].

Additionally, a large number of immunoregulatory proteins are found in AT-MSC-Exos [19,27]. AT-MSC-Exo-sourced TGF-β and NO induce cell cycle arrest and inhibit expansion of effector Th1 and Th17 lymphocytes, indoleamine 2,3-dioxygenase (IDO) promotes the generation and proliferation of immunosuppressive FoxP3-expressing Tregs, hemeoxygenase-1 (HO-1), prostaglandin E2 (PGE2), IL-10, IL-35 and IL-1 receptor antagonist (IL-1Ra) generate tolerogenic and immunosuppressive phenotype in DCs, microglia, and macrophages and create an immunosuppressive environment that inhibits inflammation in injured neural and retinal tissue [19,27].

## 3. Therapeutic Potential of AT-MSC-Exos in the Treatment of Brain and Spinal Cord Injuries

Neuronal death is the main cause of nerve function impairment [28]. Results obtained in animal models demonstrated that AT-MSC-Exos reduced neuronal apoptosis and promoted functional recovery after brain, peripheral nerve, and spinal cord injury (SCI) [29,30,31,32].

Intravenously injected AT-MSC-Exos alleviated radiation-induced brain injury by preventing apoptosis of hippocampal cells and by attenuating microglia-driven inflammation [29]. Western blot analysis of irradiated brains showed significantly lower expression of cleaved caspase-3 in hippocampal cells of AT-MSC-Exo-treated rats. Significantly reduced levels of oxidative products (malondialdehyde (MDA) and 8-hydroxydeoxyguanosine (8-OHdG)) and elevated levels of antioxidant enzymes (superoxide dismutase (SOD) and catalase (CAT)) indicated that AT-MSC-Exos managed to reduce oxidative stress in the brains of irradiated animals [29]. Additionally, AT-MSC-Exos significantly down-regulated concentrations of pro-inflammatory cytokines (TNF-α and IL-1β) and up-regulated the concentration of immunoregulatory IL-10 in irradiated brains, suggesting that AT-MSC-Exos-dependent suppression of radiation-induced inflammation was, at least partially, responsible for the beneficial effects of AT-MSC-Exos. The cellular make-up of irradiated hippocampus showed a remarkably lower number of CD68-expressing microglial cells in the brains of AT-MSC-Exos-treated rats [29]. The beneficial effects of AT-MSC-Exos in the alleviation of irradiation-induced brain inflammation relied on the immunosuppressive properties of Sirtuin 1 (SIRT-1). AT-MSC-Exos significantly increased the synthesis of SIRT-1, which inhibited the expression of Toll-like receptor 4 and prevented the NACHT leucine-rich repeat protein 3 (NLRP3)-dependent activation of the nuclear factor (NF)-κB in microglial cells. The down-regulated expression of NF-κB led to the reduced production of IL-1β in microglial cells and alleviated brain inflammation in irradiated AT-MSC-Exo-treated rats [29]. Importantly, AT-MSC-Exos-based suppression of neuro-inflammation was completely abrogated by the SIRT-1 inhibitor (EX527), indicating that anti-inflammatory and neuroprotective effects of AT-MSC-Exos relied on the activation of the SIRT-1 signaling pathway in microglial cells [29].

AT-MSC-Exos suppressed the activation of M1 microglia, alleviated neuroinflammation, prevented neuronal apoptosis, enhanced neurogenesis, and promoted functional recovery of the rats suffering from traumatic brain injury (TBI) [30]. Intracerebroventricularly injected AT-MSC-Exos accumulated in the microglia within the lesion areas. By suppressing NF-κB and mitogen-activated protein kinase (MAPK)-driven signaling in microglia, AT-MSC-Exos inhibited the production of inflammatory cytokines and chemokines (IL-1β, IL-6, TNF-α, NO, CCL2, CCL3, and CCL5) and enhanced synthesis of immunosuppressive IL-10 [30]. Accordingly, AT-MSC-Exo-dependent suppression of microglia-driven inflammation was considered mainly responsible for reduced neuronal loss and improved sensorimotor function of TBI rats [30].

AT-MSC-Exos, injected proximally and distally from the lesion site, induced neurite outgrowth and promoted functional recovery from acute sciatic nerve injury [31]. By delivering neurotrophic factors (GDNF, FGF-1, BDNF, IGF-1, NGF) directly into the injured neurons, AT-MSC-Exos supported neural survival, induced axonal re-growth, and enhanced sciatic nerve regeneration in experimental rats [31]. A significantly higher number of neurofilament-expressing axons were observed in AT-MSC-Exos-treated animals. Importantly, the sciatic function index, which gave information on the functional condition of the peripheral nerve, was significantly increased in AT-MSC-Exos-treated rats 21 days after injury, indicating that AT-MSC-Exos-based treatment led to the functional improvement of rats with peripheral nerve injury [31].

After intravenous injection, AT-MSCs were engrafted in the site of SCI and, through the delivery of trophic and angio-modulatory factors, promoted the functional recovery of experimental rats [33]. Similar to their parental cells, AT-MSC-Exos, obtained from hypoxia-conditioned AT-MSCs, significantly reduced neuronal apoptosis and improved functional recovery of the hind limbs in rats with SCI [32]. The beneficial effects of AT-MSC-Exos relied on the anti-apoptotic effects of miR-499a-5p. AT-MSC-Exo-derived miR-499a-5p modulated Jun N-terminal kinase (JNK)/c-jun-apoptotic signaling pathway and prevented apoptosis of injured neurons [32]. AT-MSC-Exos enhanced tissue repair and recovery from SCI by delivering long noncoding (Lcn) LncGm37494 RNA into inflammatory M1 microglia [32]. AT-MSC-Exos-sourced LncGm37494 induced the generation of immunosuppressive M2 phenotypes in M1 microglia by inhibiting miR-130b-3p and by promoting the expression of peroxisome proliferator-activated receptor-gamma (PPARγ), which induced the activation of transcriptional factors that were responsible for the production of anti-inflammatory cytokines (IL-10, TGF-β, IL-35) in M2 microglia [34]. Accordingly, significantly improved functional recovery of the hind limbs was observed in AT-MSC-Exos-treated SCI rats [32], indicating that AT-MSC-Exos could be considered potential new therapeutic agents for the treatment of SCI.

## 4. Beneficial Effects of AT-MSC-Exos in the Treatment of Neuroinflammatory and Neurodegenerative Diseases

Multiple sclerosis (MS) is an inflammation-mediated demyelinating disease of the central nervous system where inflammatory Th1 and Th17 cells play the most important pathogenic role [35]. By delivering cytotoxic molecules and inflammatory cytokines Th1 and Th17 lymphocytes activate M1 microglia and/or directly induce damage to neurons in the white matter of the central nervous system, resulting in the progression of neuroinflammation and demyelization [35]. Various immunomodulatory and neuroprotective factors that are able to efficiently suppress neuroinflammation are present at high concentrations in the AT-MSC-sourced conditioned medium [36]. Accordingly, AT-MSCs in a paracrine manner, through the delivery of immunoregulatory factors, induced the generation of immunosuppressive M2 phenotypes in microglia, prevented proliferation and activation of inflammatory Th1 and Th17 cells in peripheral lymph organs, and promoted the expansion of immunosuppressive, IL-10-producing Tregs, which led to the attenuation of MS in experimental animals [37]. Similar to their parental cells, AT-MSC-Exos mediated the recovery from Theiler’s murine encephalomyelitis virus (TMEV)-induced neuroinflammation, a well-established murine model of MS [38]. AT-MSC-Exos were intravenously infused 60 days after TMEV infection, once the disease was established. AT-MSC-Exos disseminated into the brain, lungs, liver, and spleen of experimental animals where attenuated Th1 and Th17 cell-driven neuroinflammation, enabling functional improvement of TMEV-infected mice. AT-MSC-Exos prevented neural cell death, enhanced cell proliferation, and reduced sizes of lesions by preventing an influx of inflammatory immune cells in the white matter of inflamed spinal cords [38]. A significantly reduced number of Iba-1-expressing microglia was observed in the brains and spinal cords of TMEV+AT-MSC-Exos-treated mice suggesting that alleviation of microglia-driven neuro-inflammation was mainly responsible for AT-MSC-Exo-based improvement of TMEV-infected animals [38]. Importantly, AT-MSC-Exos suppressed the production of pro-inflammatory cytokines that induce recruitment of circulating leucocytes in the inflamed spinal cord (TNF-α and IL-1β) and inhibited secretion of growth factors that enhance the generation and expansion of microglia and T cells (granulocyte-macrophage-colony stimulating factor (GM-CSF) and IL-2). Additionally, serum levels of Th1-related cytokines (IL-12, IL-18, IFN-γ) and Th17-associated cytokines (IL-1β, IL-6, IL-17) were significantly reduced in AT-MSC-Exos-treated TMEV-infected mice [38], suggesting that the suppression of Th1 and Th17 cell-driven inflammatory response was mainly responsible for the AT-MSC-Exo-dependent attenuation of neuroinflammation. Consequently, AT-MSC-Exos promoted the survival of neurons in the subventricular zone and improved motor function of TMEV-infected animals. The prolonged time that TMEV+AT-MSC-Exos-treated mice spent on hind legs indicated AT-MSC-Exo-dependent improvement of motor activity in AT-MSC-Exos-treated TMEV-infected animals and suggested the therapeutic potential of AT-MSC-Exos in the treatment of MS [38].

Alzheimer’s disease (AD) is a neurodegenerative disease characterized by memory loss and cognitive dysfunction [39]. The main neuropathological characteristics of AD are the formation of neurofibrillary tangles (intraneuronal aggregates of the hyperphosphorylated microtubule-associated protein tau) and the deposition of amyloid plaques (insoluble deposits of amyloid peptide). Intraneuronal aggregation of hyperphosphorylated tau proteins causes cell death of affected neurons, while the accumulation of amyloid peptides and consequent inhibition of synaptic transmission alters inter-neuronal communication [39]. AT-MSC-Exos significantly reduced the accumulation of amyloid peptides, stimulated neurogenesis, and completely restored synaptic signal transmission in the brains of APP/PS1 transgenic mice, a well-established animal model of AD [40]. AT-MSC-Exos reach the brain quickly after intranasal delivery and are mainly accumulated in neurons and glial cells. AT-MSC-Exos delivered neurotrophins (filamin-A, vinculin, neuropilin-1, neuroplastin, glia-derived nexin, flotillin-1, drebrin, teneurin-4), which induced neurogenesis, promoted myelin formation, stimulated axonal regeneration and provided protection to injured neurons [40]. AT-MSC-Exos suppressed the synthesis of pro-inflammatory cytokines in M1 microglia, which led to the alleviation of ongoing neuroinflammation. Additionally, AT-MSC-Exos raised the expression of genes that regulated synaptic transmission (*PCLO, TENM1*, and *NEXMIF*) and down-regulated the expression of the *BAD* gene, which caused cell death of injured neurons. As a result, intranasal delivery of AT-MSC-Exos reduced neurologic damage, increased the total number of newly produced neurons, and effectively corrected memory deficits in APP/PS1 mice [40].

Parkinson’s disease (PD) is a progressive neurodegenerative disease characterized by the loss of dopaminergic neurons in the substantia nigra due to increased autophagy and inflammation-induced injury [41]. Activation of NLRP3/inflammasome in microglia leads to the increased production of pro-inflammatory cytokines (IL-1β and IL-18) and results in the generation of a strong inflammatory response in the brain of patients suffering from PD [41]. In addition to neuroinflammation, cell division protein kinase 5 (CDK5)-driven autophagy is considered mainly responsible for the development and progression of PD [41]. By delivering miR-188-3p, intravenously injected AT-MSC-Exos alleviated 1-methyl-4-phenyl-1,2,4,5-tetrahydropyridine (MPTP)-induced PD in mice [42]. AT-MSC-Exos-sourced miR-188-3p directly targeted CDK5 and NLRP3 in the dopaminergic neurons and microglia. Significantly down-regulated levels of IL-1β and IL-18, the suppressed activity of NLRP3, and reduced expression of autophagy-related genes (*CDK5*, *LC3-I*, *LC3-II*, and *p62*) were observed in the serum samples and the brains of MPTP+AT-MSC-Exos-treated mice, indicating that therapeutic effects of AT-MSC-Exos on PD mice came from their ability to suppress CDK5 and NLRP3-driven autophagy and neuroinflammation in an miR-188-3p-dependent manner [42].

Amyotrophic lateral sclerosis (ALS) is a neurodegenerative disease that results in the progressive loss of motor neurons in the brain and spinal cord [43]. An increased aggregation of superoxide dismutase 1 (SOD1), decreased phosphorylation of cyclic adenosine mono phosphate-response element-binding protein (CREB) and reduced activation of peroxisome proliferator-activated receptor-γ coactivator-1α (PGC-1α) lead to the development of mitochondrial dysfunction, which is frequently observed in patients suffering from ALS [43]. Accordingly, restoration of mitochondrial function is an important therapeutic goal in the treatment of ALS [43]. Lee and colleagues showed that AT-MSC-Exos attenuated aggregation of SOD1, induced phosphorylation of CREB, and activated PGC-1α, which resulted in the restoration of mitochondrial function [44]. These results indicate that AT-MSC-Exos could be considered potential new therapeutic agents in the treatment of ALS [44].

## 5. Therapeutic Potential of AT-MSC-Exos in the Treatment of Corneal and Retinal Diseases

Results obtained in animal and clinical studies revealed that topical administration of MSCs and immunoregulatory agents (Lifitegrast, Daclizumab Rituximab, Regener-Eyes/d-MAPPS™ regenerative biologics platform technology, Adalimumab, Etanercept) efficiently attenuated inflammatory eye diseases [45,46,47].

As recently demonstrated by Shadmani and colleagues, subconjunctivally injected AT-MSCs efficiently promoted wound healing in animals that were recovering from corneal chemical burns [48]. Corneal scar and neovascularization scores were significantly lower in AT-MSC-treated rats compared to saline-treated animals with corneal injury. Histological evaluation revealed more swollen and irregular collagen bundles and a significantly higher number of inflammatory cells in the corneal stroma of saline-treated rats, suggesting that AT-MSC-based treatment managed to prevent immune cell-driven scar formation in alkali-injured corneas [48]. Immunohistochemical staining of injured corneal tissue showed significantly reduced presence of inflammatory, TNF-α, and IL-1β-producing CD68+macrophages in the corneas of AT-MSC-treated animals [49]. By suppressing macrophage-dependent, TNF-α and IL-1β-driven inflammation, AT-MSCs enabled the generation of an immunosuppressive microenvironment that supported enhanced wound healing and regeneration of injured corneal cells [49]. Additionally, AT-MSCs regulate corneal neovascularization by regulating migration and pro-angiogenic properties of cornea-infiltrated immune cells [50]. During the initial phase of corneal injury, AT-MSC enhances the influx and phagocytic properties of neutrophils which remove pathogens and damaged cells, contributing to the enhanced healing of injured tissue [50]. However, during the granulation stage of corneal regeneration, neutrophils produce various pro-angiogenic factors that induce detrimental neovascularization. Accordingly, during the granulation stage, AT-MSCs prevent neoangiogenesis by inducing transendothelial efflux of pro-angiogenic, CXCR4-expressing neutrophils from regenerated corneas [50].

Despite the fact that a large number of experimental studies demonstrated the beneficial effects of MSCs in the treatment of inflammatory diseases, there have been three cases of severe bilateral vision loss after the intravitreal injection of AT-MSCs in patients suffering from age-related macular degeneration [51]. Intravitreally administered AT-MSCs did not enter into the retinal cells and were mainly dispersed in the vitreous body. Additionally, the adherence of transplanted MSCs to the inner limiting membrane of the retina caused retinal detachment and hemorrhage, which were mainly responsible for the blindness of AT-MSC-treated patients [51].

By using the focal cranial blast model, Jha and colleagues showed that intravitreal injection of AT-MSC-sourced conditioned medium alleviated neuroinflammation, prevented oxidative-stress-induced injury, and improved survival of retinal cells in experimental animals [52]. The beneficial effects of AT-MSC-derived conditioned medium relied on the activity of immunoregulatory and neuroprotective factors that were present in AT-MSC-sourced secretome and within AT-MSC-Exos [52].

AT-MSC-Exos bind to the vitreous proteins and may remain in the vitreous body for one month [53]. The vitreous humor may act as a reserve that gradually releases AT-MSC-Exos enabling their homogeneous uptake by the injured cells in a dose-dependent manner [53].

An altered function of pericytes leads to the destabilization of the blood–retina barrier resulting in the massive infiltration of inflammatory cells in the retinas of diabetic patients [54]. AT-MSCs regulate angio-modulatory properties of pericytes and suppress the production of inflammatory cytokines in immune cells [55]. Therefore, AT-MSCs and their exosomes represent potentially new therapeutic agents in the treatment of diabetic retinopathy (DR) [56]. Results obtained in recently published studies demonstrated that AT-MSC-Exos promoted retinal tissue repair in animals suffering from streptozotocin (STZ)-induced DR [57,58]. Hyperglycemia impairs retinal microvasculature, which leads to a continuous decrease in retinal blood flow and results in the ischemic injury of retinal cells [58]. Additionally, inflammatory cytokines (TNF-α, IL-1β, IL-6) produced by CD68-expressing macrophages recruit inflammatory immune cells in the retinas of diabetic animals, crucially contributing to the development and progression of DR [58]. Massive injury of retinal cells, severe edema, extensive hemorrhage, and abnormal thickness of all retina layers (ganglionic layer (GL), inner plexiform layer (IPL), outer plexiform layer (OPL), outer nuclear layer (ONL)), observed in STZ-treated animals, were accompanied with elevated levels of TNF-α, IL-1β, IL-6 in their vitreous bodies [57,58]. AT-MSC-Exos that were subcutaneously (SC), intravenously (IV), or intraocularly (IO) injected three times (at 4, 8, and 12 weeks following STZ injection) managed to incorporate in the retinal tissue and completely restored its structure [57]. The beneficial effects of AT-MSC-Exos relied on their capacity to deliver miRNA-192 and miRNA-222 in the macrophages and ECs of injured retinas [57,58]. AT-MSC-Exos-sourced miRNA-192 inhibits the production of inflammatory cytokines in macrophages [58] while AT-MSC-Exo-delivered miRNA-222 regulates neovascularization and blood flow [57]. Accordingly, by delivering miRNA-192 and miRNA-222 in retinal macrophages and ECs, AT-MSC-Exos suppressed macrophage-driven inflammation and regulated retinal vascularization which resulted in attenuation of DR in experimental animals [57,58].

Interestingly, the therapeutic effects of AT-MSC-Exos depended on the route of their administration [57]. SC administered AT-MSC-Exos completely restored the cellular components of the retinas in diabetic animals. Retinal tissue of STZ-treated rabbits showed completely normal architecture 12 weeks after SC injection of AT-MSC-Exos [57]. Similarly, all retinal layers of diabetic animals were well-defined 12 weeks after IO application of AT-MSC-Exos. In contrast to SC and IO injected AT-MSC-Exos, IV infused AT-MSC-Exos did not manage to restore retinal architecture in diabetic mice. Irregular GL with increased thickness of all other layers was observed in the retinal tissue of STZ-treated mice 12 weeks after IV injection of AT-MSC-Exos [57]. Although IV injected AT-MSC-Exos did not restore the retinal structure, they managed to significantly alleviate hyperglycemia in STZ-treated rabbits [57]. It is well known that the majority of IV injected AT-MSC-Exos accumulate in the murine lungs, liver, and spleen which are being phagocyted by macrophages [19]. In STZ-treated diabetic animals, macrophages produce inflammatory cytokines that induce a systemic inflammatory response which leads to the increased influx of circulating leucocytes in pancreatic tissue and results in the injury of insulin-producing β cells [59]. Since AT-MSC-Exo-sourced mirmiRNA-192 suppresses the production of inflammatory cytokines in macrophages [58], the down-regulated glycemia observed in STZ-treated mice that IV received AT-MSC-Exos could be a consequence of miRNA-192 dependent suppression of macrophage-driven inflammation [57,58].

## 6. The Role of AT-MSC-Exos in Tissue Engineering and Regenerative Medicine in Neural, Retinal, and Corneal Diseases

AT-MSC-Exos have huge therapeutic potential in corneal wound healing [60]. AT-MSC-Exos induce proliferation and prevent apoptosis of corneal stromal cells (CSCs) [60]. Additionally, AT-MSC-Exos modulate the activity of MMPs and induce the synthesis of extracellular matrix (ECM)-related proteins in CSCs [60]. Significantly reduced synthesis of MMP-1, MMP-2, MMP-3, and MMP-9, but increased production of fibronectin and collagens were observed in AT-MSC-Exo-treated CSCs, indicating the therapeutic potential of AT-MSC-Exos in corneal wound healing [60].

AT-MSC-Exo-sourced NGF, BDNF, and GDNF stimulate axonal regrowth and promote the survival of injured neural and retinal ganglion cells (RGCs) [61,62,63,64]. AT-MSC-Exo-sourced NGF enhances expression of NGF-specific tropomyosin receptor kinase (Trk) A receptor enabling activation of NGF/TrkA-dependent signaling pathway in injured neural and RGCs [61,62]. AT-MSC-Exo-derived GDNF binds to the GDNF-α receptors (GDNFR). AT-MSC-Exos in NGF/TrkA and GDNF/GDNFR-dependent manner prevent apoptosis of neural cells and RGCs following ischemic or traumatic injury [61,62]. Binding of AT-MSC-Exo-derived NGF and GDNF to the TrkA and GDNFR receptors induces recruitment of Shc/Grb2 and Gap1 adapter molecules in injured neural cells, resulting in the PI3K-dependent activation of AKT kinase which phosphorylates BAD protein and Caspase-9, inhibiting their pro-apoptotic functions [63].

AT-MSC-Exo-sourced BDGF acts as neurotrophin which promotes Rac-dependent axon branching of injured RGCs [64]. Rac protein is crucially responsible for optimal RGCs’ axon arborization. The activity of Rac protein is controlled and suppressed by GTPase-activating protein (p250GAP). AT-MSC-Exo-sourced BDGF binds to TrkB receptor which results in the up-regulation of miRNA-132 that inhibits synthesis of p250GAP, enabling Rac-dependent axon branching of injured RGCs [64].

Importantly, the fact that AT-MSC-Exos, by delivering NGF, BDNF, and GDNF directly in the injured neural and retinal cells, induce prompt neuroprotection and neuritogenesis without causing severe side effects, strongly indicates that AT-MSC-Exos should be considered as potential new therapeutic agents in regenerative neurology and ophthalmology [53].

## 7. Conclusions and Future Directions

The therapeutic potential of AT-MSCs in the treatment of neurological and ocular diseases mainly relies on the activity of neuroprotective factors which are present at high concentrations in the AT-MSC-sourced secretome [65]. AT-MSC-Exos contain all AT-MSC-sourced neurotrophins, immunoregulatory and angio-modulatory factors, possess similar therapeutic potential as their parental cells, and as cell-free products overcome all safety issues related to the AT-MSC-based therapy [19,20]. However, although the biological effects of AT-MSCs and AT-MSC-Exos are very similar, signaling pathways that are elicited by AT-MSCs and their exosomes could be different [66,67]. Most recently, Garcia-Contreras and Thakor compared the cytokine profile of lipopolysaccharide (LPS)-primed microglia cells that were either co-cultured with human AT-MSCs or exposed to human AT-MSC-Exos [67]. Both AT-MSCs and AT-MSC-Exos prevented the generation of pro-inflammatory phenotypes in LPS-activated microglia cells, but molecular mechanisms responsible for their immunoregulatory effects were different [67]. While AT-MSC-Exos attenuated the production of pro-inflammatory cytokines and chemokines (IL-6, IL-8, MCP-1), AT-MSC-Exos induced the generation of immunosuppressive phenotype in LPS-primed microglia, which was documented by an increased expression of anti-inflammatory mediators (IL-10 and tissue inhibitor of metalloproteinases (TIMP)-1) [67]. In addition to divers’ mechanisms of action, AT-MSCs and their exosomes differ in stability, immunogenicity, biodistribution, and availability for immediate clinical use [66]. AT-MSC-Exos have higher stability and lower immunogenicity than their parental cells may easily bypass all biological barriers and may be readily used as an “off-the-shelf” therapeutic agent [66]. Additionally, toxicity, which could be observed as a consequence of AT-MSCs infusion, was not noticed after administration of AT-MSC-Exos, indicating that AT-MSC-Exos do not provoke similar side effects as their parental cells [66]. Although severe side effects were not reported upon infusion of AT-MSC-Exos, possible undesired effects of AT-MSC-Exo-based therapy could be related to their potent immunosuppressive properties. Secondary immunodeficiency and opportunistic infections could develop as undesirable consequences of long-term AT-MSC-Exo-based immunosuppression [68].

Results obtained in animal studies showed that AT-MSC-Exos prevented apoptotic cell death and attenuated ongoing inflammation in neural and retinal tissues (Table 2) [20,21,22,23,24,25,26,27,38,42,53,57,58]. AT-MSC-Exos play a crucially important role in the regulation of intercellular communication of injured parenchymal cells, immune cells, and ECs in the inflamed neural, corneal and retinal tissues [19]. By delivering neurotrophins, AT-MSC-Exos prevented apoptosis of injured neurons and retinal cells and promoted neuritogenesis [20,21,22,23,24,25,26,27]. In that manner, AT-MSC-Exos reduced the release of disease-associated molecular patterns (DAMPs) from injured cells and attenuated DAMPs-induced generation of inflammatory phenotype in tissue-resident immune cells [19]. Additionally, by delivering immunoregulatory factors in immune cells that infiltrated injured tissues, AT-MSC-Exos suppressed ongoing inflammation and promoted tissue repair and regeneration [19]. Finally, AT-MSC-Exos act as biological mediators that deliver pro-angiogenic miRNAs in ECs, enabling re-vascularization of ischemic neural and retinal tissues [57,58].

In line with these findings, AT-MSC-Exos have been explored as drug delivery vehicles that could efficiently transport miRNAs, recombinant proteins, and immunomodulatory drugs directly into the target cells [69]. Various chemokine receptors, lipid membrane, and nano-sized dimensions allow AT-MSC-Exos to easily bypass blood–brain and blood–retinal barriers and to home to the site of inflammation [69]. Since homing properties of AT-MSC-Exos (tropism towards particular tissue and specificity for exact target cell) could be further improved by genetic engineering of AT-MSCs, upcoming studies should focus on the design of new genetic approaches which would enhance the capacity of AT-MSC-Exo to act as drug delivery vehicles in regenerative neurology and ophthalmology [69].

There are still several issues that need to be addressed before AT-MSC-Exos could be offered as a new remedy in neurology and ophthalmology. The optimal dose, frequency, and route of AT-MSC-Exos injection should be defined for each neural and retinal disease. AT-MSC-Exos-containing eye drops efficiently alleviated NLRP3 inflammasome-driven eye inflammation and completely attenuated dry eye disease in experimental mice [70], indicating that topical administration of AT-MSC-Exos-containing eye drops could be considered a possible novel therapeutic approach for the treatment of inflammatory eye diseases, corneal and retinal injuries. Most recently, Yao and colleagues designed an MSC-Exos-containing spray that managed to restore cardiac function, reduce fibrosis and promote endogenous angiogenesis in the ischemic hearts [71]. In line with these findings, future studies could be focused on the evaluation of the therapeutic effects of AT-MSC-Exos-containing spray in the treatment of neural, corneal, and retinal diseases. The stability of AT-MSC-Exos in the spray has to be explored in future experimental studies since altered stability could minimize the therapeutic effects of AT-MSC-Exos in the treatment of neuroinflammatory and neurodegenerative diseases [71].

Upcoming studies should also determine the exact AT-MSC-Exos-containing bioactive factor(s) which is/are responsible for their beneficial effects. Administration of AT-MSC-Exos that will be enriched with the most effective neurotrophic/immunoregulatory/angio-modulatory factor(s) will enhance the therapeutic potential and efficacy of AT-MSC-Exos in the treatment of neural and retinal diseases.

Finally, AT-MSC-Exos may display different properties even when the same type of donor cells were used for their isolation [72]. Accordingly, standardization of protocols for isolation and purification of AT-MSCs will enable cost-effective, mass production of AT-MSC-Exos and will pave the way for their clinical application in neurology and ophthalmology.

## Figures and Tables

**Table 1:** AT-MSC-Exo-sourced molecules responsible for therapeutic effects of AT-MSC-Exos in the treatment of neurological and retinal diseases.

AT-MSC-Exo-Sourced Molecule(s)	Mechanism(s) of Action	Therapeutic Effect(s)	Ref.
miR-486-5p, miR-10a-5p, miR-10b-5p, miR-191-5p, miR-222-3p and miR146a	Modulation of gene expression in neural and retinal cells	Improved survival of injured neural and retinal cells	[20,21,22,23,24,25,26]
ADAM9, ADAM10	Inhibition of tissue degrading enzymes in immune cells	Enhanced regeneration of injured neuronal and retinal tissues	[27]
CACNA2D1, NOTCH2, WNT4, PAI-1	Increased survival and proliferation of injured neural cells	Enhanced neuritogenesis	[27]
MMP-2 and MMP-9	Remodeling of extracellular matrix in inflamed tissues	Enhanced regeneration of injured neuronal and retinal tissues	[27]
TP2B1, ATP1A1, PRDX-1,-2,-4,-6	Inhibited generation of reactive oxygen species in activated immune cells	Attenuation of oxidative stress in injured neural and retinal cells	[27]
GDNF, FGF-1, BDNF, IGF-1, NGF	Trophic support to the injured neurons	Enhanced axonal regeneration	[27]
TGF-β and NO	Cell cycle arrest of Th1 and Th17 lymphocytes	Reduced presence of inflammatory cells in injured neuronal and retinal tissues	[19]
IDO	Expansion of T regulatory cells	Creation of immunosuppressive microenvironment in inflamed neural and retinal tissues	[19]
HO-1, PGE2, IL-10, IL-35 and IL-1Ra	Generation of tolerogenic DCs, alternatively activated macrophages, and T regulatory cells	Attenuated neuroinflammation	[19]

**Table 2:** Animal studies demonstrating therapeutic effects of AT-MSC-Exos in the treatment of neural and retinal diseases.

Animal Model of Neural and Retinal Disease	AT-MSC-Exo-Sourced Molecule(s)	Mechanism(s) of Action	Therapeutic Effects	Ref.
radiation-induced brain injury	Sirtuin 1	attenuated activation of M1 microglia	alleviated brain inflammation and increased survival of hippocampal cells	[29]
traumatic brain injury	IL-10	suppressed activation of M1 microglia	attenuated neuroinflammation and enhanced neurogenesis	[30]
acute sciatic nerve injury	GDNF, FGF-1, BDNF, IGF-1, NGF	trophic support to injured neurons	improved survival of neural cells and enhanced sciatic nerve regeneration	[31]
spinal cord injury	miR-499a-5p	modulation of JNK)/c-jun-signaling pathway	reduced apoptosis of injured neurons	[32]
spinal cord injury	LncGm37494 RNA	inhibition of miR-130b-3p expression in M1 microglia	attenuated neuroinflammation; significantly improved functional recovery of the hind limbs	[34]
multiple sclerosis	IL-10, PGE2, TGF-β	Reduced production of inflammatory cytokines in Th1 and Th17 cells	attenuated neuroinflammation; enhanced survival of neurons; improved functional recovery	[38]
Alzheimer’s disease	filamin-A, vinculin, neuropilin-1, neuroplastin, glia-derived nexin, flotillin-1, drebrin, teneurin-4	trophic support to injured neurons	enhanced neurogenesis and axonal regeneration	[39]
Parkinson’s disease	miR-188-3p	Suppressed NLRP3-dependent generation of IL-1β and IL-18 in microglia; reduced expression of autophagy-related genes in dopaminergic neurons	attenuated neuroinflammation; enhanced survival of dopaminergic neurons	[42]
retinal injury	NGF, BDNF, GDNF	trophic support to retinal cells	enhanced neuritogenesis; reduced injury of retinal cells	[53]
diabetic retinopathy	miRNA-192 and miRNA-222	Suppressed synthesis of inflammatory cytokines in macrophages; Modulated synthesis of vasoactive factors in endothelial cells;	attenuated inflammation; regulated retinal vascularization	[57,58]

## Data Availability

The data presented in this study are available on request from the corresponding author.
